# Functional transplantation of salivary gland cells differentiated from mouse early ES cells in vitro

**DOI:** 10.1007/s13577-013-0061-z

**Published:** 2013-05-17

**Authors:** Miyuki Kawakami, Hiroshi Ishikawa, Toshiaki Tachibana, Akira Tanaka, Izumi Mataga

**Affiliations:** 1 Department of Oral and Maxillofacial Surgery, School of Life Dentistry, The Nippon Dental University, Niigata, Japan; 2 Department of NDU Life Sciences, School of Life Dentistry, The Nippon Dental University, Tokyo, Japan; 3 Department of Anatomy, The Jikei University, School of Medicine, Tokyo, Japan; 4 Department of Oral and Maxillofacial Surgery, Niigata Hospital, The Nippon Dental University, Niigata, Japan; 5 Division of Cell Regeneration and Transplantation, Advanced Research Center, School of Life Dentistry, The Nippon Dental University, Niigata, Japan

**Keywords:** Salivary gland, Cell-based therapy, Co-culture system, Cell differentiation, Regeneration

## Abstract

Atrophy or hypofunction of the salivary gland because of aging or disease causes hyposalivation and has an effect on the quality of life of patients, for example not only dry mouth but deterioration in mastication/deglutition disorder and the status of oral hygiene. Currently conducted therapies for atrophy or hypofunction of the salivary gland in clinical practice are only symptomatic treatments with drugs and artificial saliva, and therefore it is preferable to establish a radical therapy. At this time, as a fundamental investigation, by co-culturing mouse early ES (mEES-6) cells with human salivary gland-derived fibroblasts (hSG-fibro), differentiation of mEES-6 cells to salivary gland cells has been attempted. Also, the possibility of cell engraftment was examined. After identifying the cells which were co-cultured with GFP-transfected mEES-6 cells and hSG-fibro, the cells were transplanted into the submandibular gland of SCID mice, and the degree of differentiation into tissues was examined. The possibility of tissue functional reconstitution from co-cultured cells in a three-dimensional culture system was examined. Our results confirmed that the co-cultured cells expressed salivary gland-related markers and had an ability to generate neo-tissues by transplantation in vivo. Moreover, the cells could reconstitute gland structures in a three-dimensional culture system. By co-culture with hSG-fibro, mEES-6 cells were successfully differentiated into salivary gland cells which were transplantable and have tissue neogenetic ability.

## Introduction

The cause of atrophy or hypofunction of the salivary gland mainly includes radiotherapy for head and neck cancer, obstructive defect in ducts, chronic graft-versus-host disease (GVHD), following bone-marrow transplant, or age-related change. In these conditions, the salivary gland, especially the acinar cells, are remarkably impaired, and atrophy or a decrease in the cells has been recognized, leading to a loss of functional parenchymal tissue. It is known that this status causes decreased saliva secretion (i.e. dry mouth), significantly effecting the quality of life (QoL) of patients, such as deterioration in mastication/deglutition disorder and the status of oral hygiene [1, 2]. Even though there are many patients with dry mouth, currently conducted therapies for it caused by atrophy or hypofunction of the salivary gland in clinical practice are only symptomatic treatments, including transiently increasing secretory capacity of residual acinar cells by drug administration or moisturizing dry mouth tissues by using artificial saliva, and less invasive radical therapy to improve the QoL of patients has not yet been established [3, 4].

In this study, we examined methods of differentiation into salivary gland cells from stem cells with only biomaterials, targeting the development of a less invasive radical therapy for dry mouth by regeneration of salivary gland cells. For biomaterials, mouse-derived early ES (mEES-6) cells [5] were used as the cell source, while fibroblasts were used as an inducer, which have been suggested as possibly having organ-specific characteristics. We assumed that fibroblasts could be procured under given culture conditions even though the degree of atrophy varies among the salivary gland tissues to be collected, and that the organ-specific characteristics could induce collected organ-derived cells. The prospects seem promising to induce stem cells in salivary gland cell differentiation by co-culture with salivary gland-derived fibroblasts in order to prompt the generation of neo-salivary gland tissue and to finally restore the gland function by transplantation of the differentiated salivary glands cells in vivo. At this time, as a fundamental investigation, we examined whether mEES-6 cells, in which establishment has been previously reported, can be differentiated into salivary gland cells, and whether those cells are transplantable in vivo.

## Materials and methods

### Animals

In transplant experiments, submandibular glands of 8-week-old female SCID/Jcl mice (CLEA Japan, Tokyo, Japan) were used. While the mice were under intraperitoneal anesthesia by pentobarbital sodium (50 mg/kg ip), the following transplantation of cultured salivary gland cells and the extirpation of transplanted tissues were performed. Methods for animal husbandry and slaughter conformed to the Code of ethics for experimental animals of The Nippon Dental University School of Life Dentistry at Niigata.

### Culture materials and methods

#### Cells used

1. Mouse-derived early ES (mEES-6) cells

As a cell source, mEES-6 cells established from 2-cell-stage mouse embryos were used. The mEES-6 cells-produced chimera mouse was used in this study [5]. At this time, in order to identify whether the tissues are derived from mEES-6 cells, when the transplanted tissue in vivo forms new tissue, mEES-6 cells induced from GFP (promoter: CMV; vector: pE GFP; Clontech, Palo Alto, CA, USA) were used [6]. Whether or not mEES-6 cells used in the experiment maintain the pluripotency and undifferentiated state was confirmed by teratoma formation through cell transplantation to SCID mice. mEES-6 cells [approximately 1 × 10^7^ cells/0.5 ml (Hanks’ solution)] were injected subcutaneously into the dorsal neck of a mouse.

2. Human salivary gland-derived fibroblast (hSG-fibro)

After obtaining informed consent from patients, some normal tissues were collected during salivary gland cancer surgery. The collected normal salivary gland tissues were washed with Hanks’ solution, sliced with a razor-type scalpel, and the strips of the slices were cultured under static conditions. The outgrowing fibroblasts were separated by colonial cloning by a filter paper method [6], and applied to experiments after identifying them using immunostaining and RT-PCR.

#### Cell culture methods

The cells described above were cultured in a growth medium (GM): DMEM/F12 supplemented with 15 % fetal bovine serum (FBS), 10 μM non-essential amino acids solution, 50 U/ml penicillin and 50 μg/ml streptomycin, and 0.25 μg/ml Fungizone (all Life Technologies, CA, USA) within a CO_2_ incubator (4.7 % CO_2_ + 95.3 % air). The GM was exchanged twice a week. In addition, leukemia inhibitory factor (LIF; 1 ng/ml' Wako Pure Chemical Industries, Osaka, Japan) was added to the culture medium to maintain an anaplastic status of the mEES-6 cells according to our previous reports [7–9]. During culture of the cells, the cells were observed using an inverted-phase contrast microscope (Olympus, Tokyo, Japan). A solution of 0.2 % trypsin–0.02 % EDTA/PBS(−) (Trypsin 250; Difco, Detroit, IL, USA) and Hanks’ solution (Nissui, Tokyo, Japan) was used for primary culture and subcultures. Cells were cultured in 60-mm dishes (Falcon Plastics, Franklin Lakes, NJ, USA). Whole manipulations in cell culture (primary culture, subculture, cryopreservation) were accomplished using 5-ml disposable pipettes (NIPPON Genetics, Tokyo, Japan) and 15-ml (Greiner-bio-one, Tokyo, Japan) and/or 50-ml centrifugal tubes (Falcon Plastics).

### Induction to salivary gland cells using co-culture system (co-SG cells)

In a co-culture system with mEES-6 cells and hSG-fibro, co-SG cells were induced. The co-SG cells mean the salivary gland cells derived from co-culture with mEES-6 cells and hSG-fibro.

Before co-culture, the growth potential of the hSG-fibro was preliminarily eliminated by treating with mitomycin C (10 μg/ml; Kyowa Hakko Kirin, Tokyo, Japan) containing GM according to the previous report of cultured salivary gland cells [10]. After the treatment, the hSG-fibro was sufficiently washed using Hanks’ solution, and co-cultured by seeding mEES-6 cells. In the co-culture, LIF-free GM was used, and the medium was exchanged twice a week. In addition, no materials having a differentiation-inducing effect were used. The cultured cells were used within three passages of culture for analysis and experiments.

### General staining (HE staining, PAS staining), and immunofluorescent and histochemical staining

#### Tissue staining

While extirpating tissues of mice, thoracotomy was performed under general anesthesia. After perfusion using Hanks’ solution, perfusion fixation was performed with 4 % paraformaldehyde (PFA). The submandibular glands were extirpated, embedded in a fixing solution and then were embedded in paraffin after dehydration. The tissue sections were prepared at a 4-μm thickness and applied to staining. In histological analysis, HE staining and Periodic Acid Schiff’s base (PAS) staining were performed. 

Immunostaining was performed by using the following protocol after deparaffinization of sections. After washing with PBS, samples were activated in 0.1 % trypsin at 37 °C for 30 min and blocked with 1 % bovine serum albumin at room temperature for 30 min. Subsequently, they were incubated with a primary antibody at 4 °C overnight, washed with PBS, and incubated with a secondary antibody (diluted 1:1,000) at room temperature for 2 h. For nuclear staining and inclusion, a Vectashield mounting medium with DAPI (Vector Laboratories, Burlinghame, CA, USA) was used.

#### Cell staining

Part of the cells from hSG-fibro and co-SG cells during passage were seeded in Laboratory-Tek II chamber slides (Nalge Nunc, Roskilde, Denmark), cultured in a serum culture, and stained for 3–4 days after seeding. The cells were stained according to the above protocol except for the activation step, followed by fixing cells with 100 % methanol (Wako Pure Chemical Industries, Osaka, Japan) at −30 °C for 10 min.

Primary antibodies used in this experiment are shown in Table 1.

**Table 1:** List of primary antibodies

Antibody	Company (catalog no.)	Dilution
Anti-Vimentin	Sigma-Aldrich (V6630)	1:1,000
Anti-mitochondria	Millipore (#MAB1273)	1:1,000
Anti-GFP	Aves Labs (GFP-1010)	1:500
Anti-amylase	Sigma-Aldrich (A8273)	1:100
Anti-basic FGF	Abcam (ab65973)	1:1,000
Anti-NGF	Abcam (ab49205)	1:250
Anti-cytokeratin	Dako (M3515)	1:1,000

### Analysis by RT-PCR

hSG-fibro and co-SG cells were used as samples.

In the analysis by RT-PCR of this experiment, we examined how the induction of differentiation by co-culture effects the changes in gene expression of hSG-fibro (inducer) and mEES-6 cells (cell source). Because the cells sourced from heterologous individuals are used in co-culture, differences in gene expression due to co-culture were examined before and after induction of differentiation, by confirming gene expression utilizing specificity of primers to distinguish among individuals. The used primers were confirmed to have no cross-reactivity between human and mouse (data not shown).

Total RNA was extracted and purified from each cell using an RNeasy mini kit (QIAGEN, Hilden, Germany). Using 1 μg mRNA of that, cDNAs were synthesized using a high-capacity cDNA Reverse Transcription kit (Life Technologies). In the PCR amplification of cDNA, Platinum PCR SuperMix and Veriti™ Thermal Cycler (both Life Technologies) were used. The conditions of PCR are as follows: an initial denaturing step of 95 °C for 2 min, 35 repetitive cycles of denaturing at 95 °C for 30 s, primer annealing at 54–58 °C for 30 s and an extension at 72 °C for 1 min, and then a final extension at 72 °C for 10 min.

The PCR products were all separated via gel electrophoresis (2 % gel) and visualized via UV detection using EtBr. The sequences of the primers used in this experiment are shown in Table 2. For internal control, glyceraldehydes-3-phosphate dehydrogenase (GAPDH) was used. The above experimental assay kits were used according to each manufacture’s instructions.

**Table 2:** Sequences of primers

Target gene	Primer sequence (5′–3′)	GenBank accession no.
Human primers		
Vimentin	F:GGGACCTCTACGAGGAGGAG	NM_003380
	R:CGCATTGTCAACATCCTGTC	
Collagen type1	F:CCAAATCTGTCTCCCCAGAA	NM_000088
	R:TCAAAAACGAAGGGGAGATG	
α-Amylase1	F:AATACACAACAAGGACGGACATC	NM_000690
	R:TCCAAATCCCTTCGGAGCTAAA	
AQP-5	F:CGGGCTTTCTTCTACGTGG	NM_001651
	R:GCTGGAAGGTCAGAATCAGCTC	
bFGF	F:AGAAGACGACCCTCACATCA	NM_002006
	R:CGGTTAGCACACACTCCTTTG	
NGF	F:GGCAGACCCGCAACATTACT	NM_002506
	R:CACCACCGACCTCGAAGTC	
PSCA	F:TGCTTGCCCTGTTGATGGCAG	NM_005672
	R:CCAGAGCAGCAGGCCGAGTGCA	
Sox-2	F:CCCCCGGCGGCAATAGCA	
	R:TCGGCGCCGGGGAGATACAT	
Nanog	F:CAGCCCCGATTCTTCCACCAGTCCC	
	R:CGGAAGATTCCCAGTCGGGTTCACC	
Oct3/4	F:GACAGGGGGAGGGGAGGAGCTAGG	
	R:CTTCCCTCCAACCAGTTGCCCCAA	
GAPDH	F:GAGTCAACGGATTTGGTCGT	NM_002046
	R:TTGATTTTGGAGGGATCTCG	
Mouse primers		
Vimentin	F:CGTCCACACGCACCTACAG	NM_001701
	R:GGGGGATGAGGAATAGAGGCT	
Collagen type 1	F:GTAACTTCGTGCCTAGCAACA	NM_007743
	R:CCTTTGTCAGAATACTGAGCAGC	
α-Amylase1	F:GACGAACTGCTATTATCCACCTG	NM_007446
	R:GTTGCACCTGTTCACCATGTC	
AQP-5	F:AGAAGGAGGTGTGTTCAGTTGC	NM_009701
	R:GCCAGAGTAATGGCCGGAT	
bFGF	F:GAGTTGTGTCTATCAAGGGAGTG	NM_008006
	R:CCGTCCATCTTCCTTCATAGC	
NGF	F:AAGCCCACTGGACTAAACTTCA	NM_001112698
	R:GGGCAGCTATTGGTGCAGTA	
PSCA	F:GGACCAGCACAGTTGCTTTAC	NM_028216
	R:GTAGTTCTCCGAGTCATCCTCA	
Sox-2	F:GCGGAGTGGAAACTTTTGTCC	NM_011443
	R:GGGAAGCGTGTACTTATCCTTCT	
Nanog	F:TCTTCCTGGTCCCCACAGTTT	NM_028016
	R:GCAAGAATAGTTCTCGGGATGAA	
Oct3/4	F:AAAAAGCAGGCTCCACCTTCCCCATGGCTGGACACC	NM_013633
	R:AGAAAGCTGGGTTGATCAACAGCATCACTGAGCTTC	
GAPDH	F:TGATGACATCAAGAAGGTGGTGAAG	NM_008084
	R:TCCTTGGAGGCCATGTAGGCCAT	

### Electron microscopic observation

The cells in a 35-mm dish were fixed with 2.5 % glutaraldehyde in 0.1 M phosphate buffer for 1 h at room temperature and post-fixed with 1 % OsO_4_ in the same buffer at 0 °C for 30 min. The specimens were dehydrated with ethanol, immersed twice in absolute propylene oxide, and embedded in Quetol 812. Sections were cut at a thickness of about 80 nm with a diamond knife. Following staining with uranyl acetate and lead citrate, the specimens were observed with a JEOL JEM-1200EX-II electron microscope at 80 kV.

### co-SG cells transplantation in vivo

Co-SG cells were transplanted into normal submandibular glands of mice (Jcl scid/scid 8-week-old, female) to examine whether co-SG cells induced differentiation in co-culture using GFP-transfected mEES-6 cells can be engrafted in vivo, and whether or not the cells can be differentiated to a salivary gland in vivo. Under anesthesia, an incision was made in the submandibular region, and co-SG cells (1 × 10^5^ cells/0.2 ml Hanks’ solution) were megascopically injected into the submandibular glands. For the injection, a 23-G needle was used. Mice were euthanized at around 1 month after transplantation, and the transplanted submandibular glands were extirpated.

### Reconstitution of salivary gland tissues

Reconstitution of tissues was examined in a three-dimensional culture system to examine whether the salivary gland-like tissue structures can form in vitro. For tissue reconstitution, mEES-6 cells and hSG-fibro were co-cultured three-dimensionally in a collagen sponge (type I collagen sponge; Stem, Tokyo, Japan). The culture method is as follows: hSG-fibro was injected into a collagen sponge which was sufficiently soaked in a medium. After being cultured under static conditions for 2 weeks, the cells were colonized. The salivary gland cells which were differentiated in co-culture with mEES-6 cells and hSG-fibro were injected into a collagen sponge colonized with hSG-fibro. After being cultured under static conditions for 1 week, the sponge was transferred to the Rose chamber for circumfusion culture, and circumfusion culture (6 ml/min) was performed for approximately 3 weeks. The medium (200 ml/bottle) was changed every 3 days. Cultured constructs were examined by histological analysis.

## Results

### Cultured cells analysis

#### mEES-6 cells

**Fig. 1:**
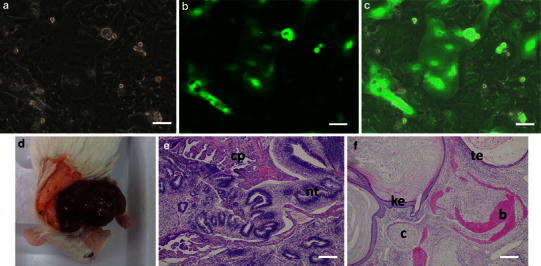
Confirmation of mEES-6 cells characteristics. **a** Phase-contrast micrograph. **b** Fluorescence micrograph. **c** Merged images of phase-contrast micrograph and fluorescence micrograph. Micrographs of (**b**, **c**) stained with antibody. **d**–**f** Tissue formation after transplantation of mEES-6 cells to mouse submandibular gland. **d** Macro-photograph. **e**, **f** H&E staining, *ke* keratotic stratified squamous epithelium, *te* tracheal epithelium, *b* bone, *c* cartilage, *cp* choroid plexus, *nt* neural tube. *Scale bars* 50 μm

First, mEES-6 cells during culture were observed under a fluorescence microscope and confirmed that GFP was expressed in all cells (Fig. 1a–c). Next, whether or not mEES-6 cells maintain the pluripotency was confirmed by teratoma formation through the mEES-6 cells transplantation into mice (Fig. 1d–f). An elastic soft tumor developed at the transplant site approximately 1–2 months after transplantation of mEES-6 cells into mice. Results of HE staining confirmed it to be a teratoma composed of tridermal tissues such as bone, cartilage, skin, tracheal epithelium, and neural tube (neural anlage). These results confirmed that mEES-6 cells maintained their pluripotency.

#### hSG-fibro

**Fig. 2:**
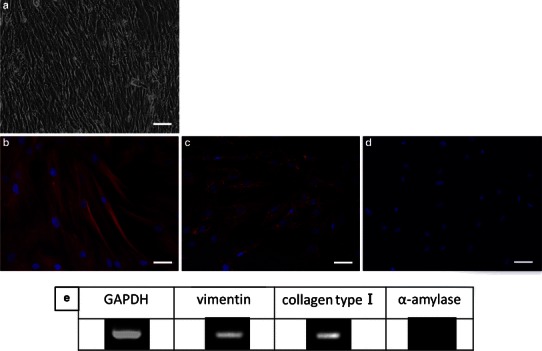
Confirmation of hSG-fibro characteristics. **a** Phase-contrast micrograph. **b**–**d** Immunostaining image: **b** vimentin (*red*), **c** human-specific mitochondria (*red*), **d** amylase (*red*). Cell nuclei depicted with DAPI staining (*blue*). *Scale bars* 50 μm. **e** RT-PCR analysis

The cultured fibroblast showed a typical spindle-shaped morphology in phase-contrast micrograph (Fig. 2a). Further analysis by immunostaining of cells (Fig. 2b, c) and RT-PCR (Fig. 2e) revealed that they were human-derived fibroblast. Notably, in the results of the analysis of the amylase by RT-PCR and immunostaining the expression was not observed. In other words, the cell population was isolated from the salivary glands, and it was confirmed that it does not include any acinar cell components (Fig. 2d, e).

### Induction to salivary gland cells using co-culture system (co-SG cells)

**Fig. 3:**
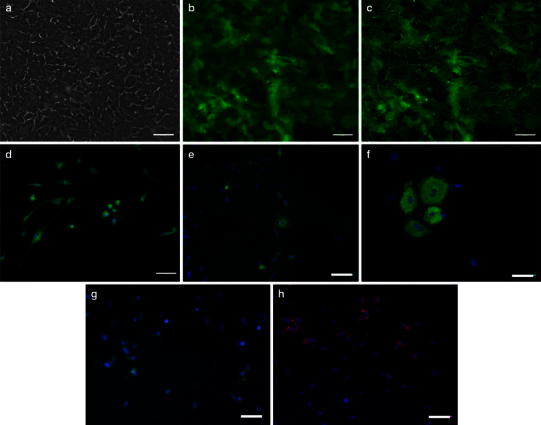

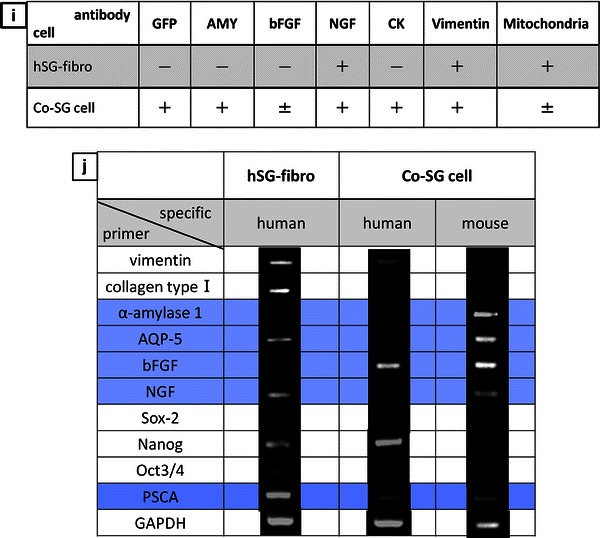
Confirmation of cells characteristics after co-culture with mEES-6 cells and hSG-fibro. **a** Phase-contrast micrograph. **b** Fluorescence micrograph. **c** Merged images of phase-contrast micrograph and fluorescence micrograph. Micrographs of (**b**, **c**) stained with antibody. **d**–**h** Immunostaining image: **d** green fluorescent protein (GFP), **e** amylase, **f** cytokeratin (CK), **g** basic fibroblast growth factor (bFGF), **h** nerve growth factor (NGF). Cell nuclei depicts with DAPI staining (*blue*). *Scale bars* 50 μm. **i** A summary of the immunofluorescence results indicating positive (+) or negative (−). **j** Confirmed changes in gene expression by RT-PCR before and after co-culture

There was an obvious change in the morphology of the cultured cells approximately 1 week after co-culture with the mEES-6 cells and hSG-fibro (Fig. 3a), compared to the cell morphology during the mEES-6 cells culture (Fig. 1a). Expression of GFP was confirmed in almost of all cells, and indicated that it played a role as cell source (Fig. 3b, c).

These cells were characterized by immunostaining (Fig. 3d–h) and RT-PCR (Fig. 3j, co-SG cells). Given the results demonstrating that salivary gland-related markers such as amylase, AQP-5, bFGF and NGF were expressed in the cells, they had similar characteristics to the salivary gland. In addition, when differences in expressed proteins in the cells were compared before and after induction of differentiation through co-culture, the results of both immunostaining and RT-PCR showed certain changes in the expressed proteins (Fig. 3i, j). Notably, when changes in gene expression were compared before and after induction of differentiation by RT-PCR analysis, expression of AQP-5 and NGF disappeared from the hSG-fibro and appeared in the mEES-6 cells after co-culture. In contrast, the expression of Amylase and bFGF appeared by co-culture even though there was no expression before the co-culture. Thus, these results confirmed that the genes expressed in each cell changed before and after the co-culture, and that their characteristics changed.

### Transplantation of co-SG cells in vivo

**Fig. 4:**
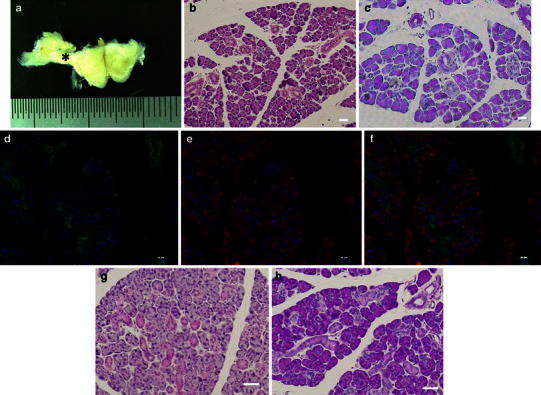
Cell transplantation of cultured salivary gland cells to normal submandibular glands of mouse. **a** Macro-photograph; cell-transplanted side is seen in the *left* of the photograph. The tissue enlargement area after transplantation (*asterisk*). **b**–**f** Histological analysis of area with *asterisk*: **b** H&E staining. **c** PAS staining. Immunostaining image (**d**), green fluorescent protein (GFP), (**e**) amylase, (**f**) merge image. **g**–**i** Normal (non-treatment) salivary gland: **g** H&E staining. **h** PAS staining. Cell nuclei depicts with DAPI staining (*blue*). *Scale bars* 50 μm

After confirming the cells obtained from the co-culture with mEES-6 cells and hSG-fibro described above were salivary gland cells, the cells were transplanted to a normal submandibular gland of a mouse. One month after transplantation, obvious tissue enlargement in the cell-transplanted side of the submandibular gland was observed compared to the non-transplanted side (Fig. 4a). When the enlarged area of tissue was examined histologically by HE staining and PAS staining, observations almost similar to a normal submandibular gland, such as a structure of acinar and duct and lobular formation, were confirmed (Fig. 4b, c). The enlarged area of tissue was composed of all of the GFP-positive cells (Fig. 4d) and the acinar cells showed amylase-positive (Fig. 4e). These results showed that transplantation of co-SG cells which were induced by the co-culture with mEES-6 cells and hSG-fibro into normal tissues in vivo leads to the regeneration of neo-salivary gland tissues that may produce amylase and have a functional role (Fig. 4d–f). For a comparison, we show the normal (non-treatment) mouse salivary gland tissue (Fig. 4g, h).

### Reconstitution of salivary gland tissues

**Fig. 5:**
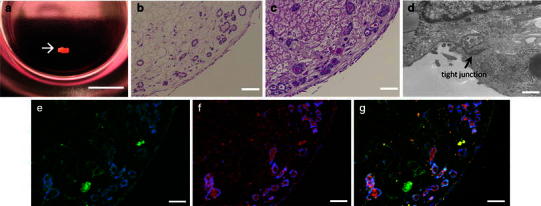
Reconstitution of salivary gland tissues by three-dimensional culture. **a** Macro-photograph. Cultured construct (*arrow*), *scale bar* 5 mm. **b** H&E staining. **c** PAS staining. **d** Transmission electron micrograph, *scale bars* 500 nm. **e**–**g** Immunohistological staining: **e** green fluorescent protein (GFP), **f** amylase, **g** merge image. Cell nuclei depicts with DAPI staining (*blue*). *Scale bars* 50 μm

Samples obtained from co-SG cells which were reconstituted by a three-dimensional culture were examined by histological analysis (Fig. 5a–c). An HE staining image confirmed that a acinar-like or duct-like structure was formed inside a sponge. The results of immunostaining confirmed that the formed constructs were GFP-positive cells, that is, they were composed of mEES-6 cells (Fig. 5e). Furthermore, the results of PAS staining demonstrated that the inside of the duct-like structure was positive, and it could be specified that it was a substance of amylase-positive based on the immunostaining of the same area (Fig. 5f). These results confirmed that the co-SG cells which were induced by co-culture with mEES-6 cells and hSG-fibro could form acinar-like or duct-like structures which produce amylase by three-dimensional culture in a sponge (Fig. 5e–g). However, the formation of duct-like structure had been confined to the peripheral area of the sponge (Fig. 5b, c). Furthermore, a transmission electron micrograph (Fig. 5d) showed the formation of a junctional complex, suggesting that communication among cells constituting a duct-like structure may be established.

## Discussion

If transplantation of salivary gland cells which are functionally differentiated from stem cells in a culture into salivary glands with atrophy or hypofunction because of aging or disease can help to regenerate solid organs including salivary gland tissues, especially the acinar and duct system, restoration of salivary gland function with atrophy or hypofunction or radical therapy for dry mouth may potentially be realized. From a clinical standpoint, transplantation of salivary glands tissues which are three-dimensionally constituted in a culture, which may facilitate regeneration and substitution of neo-salivary glands by cell transplantation, may be less invasive in vivo and a highly probable technique.

Recently, various investigations such as gene therapy, tissue engineering and cell-based therapy have proceeded towards the establishment of regenerative medicine for the salivary glands. Until now, two main regenerative approaches to functional recovery of the salivary gland have been developed.

The first is an approach to constitute an artificial salivary gland using cultured salivary gland epithelial cells by applying tissue engineering [11, 12]. However, in this approach, only ductal cells could be regenerated, whereas functional regeneration of salivary gland tissues (acinar tissues) was difficult. The second is an approach with a tissue stem cell transplantation treatment. In recent years, studies using bone marrow-derived stem cells have been performed. These studies have reported that in vitro, these cells can differentiate to epithelial cells [13–15], and that, in vivo, the transplanted cells stay in tissues and possibly have a salivary gland function by transplantation of stem cells, which are isolated from the salivary gland to atrophic salivary gland with damage [16, 17]. In previous studies, the results showed that ductal cells with several stem cell markers positively exist in the ductal compartment of salivary glands [18, 19] and that acinar cells were regenerated by cell transplantation of those cells to the salivary gland of an irradiated mouse model, which resulted in recovery of the saliva secretion [20]. However, for these stem cells, salivary gland-derived cells should be used, and the isolation of stem cells from the salivary gland with a markedly impaired function may be difficult. Therefore, other methods separating normal salivary gland-derived glandular epithelial cells aside from stem cells and transplanting the cells have been investigated. However, in these transplant experiments, the isolated cells might not have engrafted into the nearly normal salivary gland tissues [13]. Therefore, reengineering of the graft cells with a higher regenerative ability is expected.

In consideration of applying these approaches clinically, a sufficient number of stem cells for regenerative therapy might not be secured, because the target tissues for cell isolation are from already atrophied salivary glands due to disease or aging, In the isolation of stem cells, culture conditions should be researched and set individually depending on the collected tissue status, suggesting that this method cannot be applied so easily to clinical settings.

In the present study, we examined methods for inducing functional differentiation of salivary gland cells from mEES-6 cells by co-culture with mEES-6 cells and hSG-fibro, targeting easy reengineering of graft cells with a higher regenerative ability. Transplantation of salivary gland cells which are differentiated from mEES-6 cells to within normal submandibular glands of mice led to the generation of neo-salivary gland cells. These results demonstrated that differentiation was induced by co-culture. Moreover, unlike the above report, transplantation of salivary gland cells differentiated to a normal submandibular gland, resulting in the generation of neo-salivary gland cells, which can be engrafted. These data suggested that the salivary gland cells differentiated by our culture system used in this study were engraftable, and that the tissues are regenerable without the possibly of inducing a cell inhibition mechanism in vivo [21].

In the analysis of the cultured salivary gland cells, the results showed that gene expression was different before and after induction of cell differentiation. This suggests that there is an interaction between other factors, and that each factor expresses sequentially during induction of differentiation to salivary gland cells. The mesenchymal–epithelial interactions are very important for the differentiation and/or development during embryogenesis [22]. Furthermore, certain factors in fibroblasts might have autoregulation that serves as a trigger for the onset of induction of differentiation, and the offset of the production of factors once the direction to differentiation of mEES-6 cells is fixed. The secreted proteins from the salivary gland such as amylase and bFGF were not expressed by hSG-fibro alone; however, they were expressed after infusion of differentiation of salivary gland cells. However, the analysis performed this time was for fragmentary and portions of factors. Therefore, in the self-tissue regeneration of salivary gland cells, it is necessary to perform further analysis of the growth factors in salivary gland cells (especially fibroblasts) and the interaction of cytokines, or the difference in the level of expression of the factors due to time-dependent changes in the differentiation process. If the difference of factors due to the time-dependent changes in the differentiation process of salivary gland cells becomes clear, detailed conditions in culture and differentiation for graft cells can in the future be set towards the application of salivary gland cells transplantation to humans.

Furthermore, in regard to tissue reconstitution by a three-dimensional culture system, the differentiated salivary gland cells in the co-culture system in this study showed constructability of salivary gland-like tissues in vitro. The cells in vitro also showed higher tissue regenerability. In the future, when the accuracy of constructs is enhanced by improvement of culture techniques, transplantation of tissues to salivary gland loss might be possible. For this purpose, at this point, we will examine if salivary gland-like tissues which are constituted by three-dimensional culture maintain their stability in vivo.

In recent research on salivary gland regeneration, research on cell transplantation using bone marrow-derived stem cells is attracting a lot of attention, and reporting relatively good results [23–25]. However, great challenges to human application still remain, such as (1) even though the quantity of cells which are isolated from stem cells as a cell source is sufficient, the number of engrafted cells is limited, or the tissue regeneration fails if a certain degree of function is not remaining in the donor cells, due to a certain mechanism of cell adhesion inhibition in vivo; and (2) the difficulty of harvesting bone marrow. Our investigation is proceeding into the application of human tissue stem cells by procuring better graft cells in order to apply themmore easily to clinical settings in the future.

## Conclusion

In our study, salivary glands cells which are differentiated by co-culture with mouse early ES cells and human salivary gland-derived fibroblasts are the most useful approach to establish a radical therapy for atrophy or hypofunction of the salivary gland and dry mouth.
